# Acceleration of genetic gain in cattle by reduction of generation interval

**DOI:** 10.1038/srep08674

**Published:** 2015-03-02

**Authors:** Poothappillai Kasinathan, Hong Wei, Tianhao Xiang, Jose A. Molina, John Metzger, Diane Broek, Sivakanthan Kasinathan, David C. Faber, Mark F. Allan

**Affiliations:** 1 Trans Ova Genetics, LC, Sioux Center, IA 51250; 2 Medical Scientist Training Program, University of Washington School of Medicine, Seattle, WA 98195

## Abstract

Genomic selection (GS) approaches, in combination with reproductive technologies, are revolutionizing the design and implementation of breeding programs in livestock species, particularly in cattle. GS leverages genomic readouts to provide estimates of breeding value early in the life of animals. However, the capacity of these approaches for improving genetic gain in breeding programs is limited by generation interval, the average age of an animal when replacement progeny are born. Here, we present a cost-effective approach that combines GS with reproductive technologies to reduce generation interval by rapidly producing high genetic merit calves.

GS strategies can theoretically shorten generation interval, increase prediction accuracy and selection intensity, reduce cost of breeding programs, and allow evaluation of traits that have phenotypes that are difficult to evaluate^1^,^2^,^3^,^4^. This potential of GS to improve bovine breeding programs is of substantial interest given the increasing global demand for dairy and meat production^5^. The largest increase in genetic gain can be achieved by shortening the generation interval^6^. In principle, genetic gain can also be improved by employing GS strategies in combination with advanced reproductive technologies. Single nucleotide polymorphism (SNP)-based enhanced genotyping can improve the accuracy of selection, while multiple ovulation, embryo transfer, and *in vitro* fertilization (IVF) can improve the intensity of selection. Importantly, genotyping cells obtained from biopsy of pre-implantation embryos can substantially shorten the generation interval^2^. While pre-implantation stage embryo production and selection is much less expensive than traditional breeding and selection, implementing selection at the embryo stage has several limitations including technical challenges in embryo biopsy, quality of the isolated DNA from the biopsy for whole genome amplification (WGA), and the possible impact of freezing on developmental potential of embryos^6^,^7^,^8^. Moreover, WGA from a limited number of embryonic cells can result in excessive allele dropout rate, which leads to lower SNP call rates relative to the threshold standards needed for genomic enhanced genetic analysis^9^,^10^.

In order to address the limitations of current approaches to reduce generation interval, we evaluated a new method for production of high genetic merit calves, which combines advanced reproductive processes such as multiple ovulation embryo transfer, ovum pick-up, IVF, genomic analyses at early an embryonic stage, and somatic cell nuclear transfer (SCNT) cloning (Fig. 1a). This approach is scalable and can lead to considerable savings for breeders by achieving substantial reduction in generation interval and selectively producing animals with the desired genetics within a timeframe of approximately one year.

In order to increase the number of selection candidates, elite Jersey females were stimulated with follicle stimulating hormone followed by ovum pick-up in combination with IVF to produce embryos. Depending on the requirement, sexed or conventional Jersey semen was used for IVF. We carried out multiple embryo transfers (5–8 embryos) per recipient female to minimize expenses involved with embryo transfer and flushing. We collected fetuses between 21–26 days of gestation for genomic analysis. First, we compared the fetal recovery rate at flushing during 21–23 and 24–26 days of gestation. Fetal recovery rate at flushing was similar between 21–23 days of gestation compared to the recovery rate at 24–26 days of gestation (Table 1); however, fetuses collected between 24–26 days were not intact, leading to possible duplication of fetuses for cell line establishment and genomic analysis. Therefore, fetuses collected between 21–23 days of gestation were used to generate individual cell lines.

Individual fibroblast cell lines were established from flushed early stage embryos with a high success rate (97.3%, Table 1). Cell lines were passaged once and cultured for 3–5 days in order to obtain homogenous fibroblast populations. DNA isolated from fibroblast cell lines was submitted for genomic enhanced genotyping using low-density beadchip. The average SNP call rate for all 71 cell lines was high (99.2 ± 0.001%). Genotype results were submitted to the Council on Dairy Cattle Breeding for assessment of enhanced genetic merit values for economically important traits in dairy cattle.

We selected one cell line (designated FL065) based on values of the general Jersey performance index and other relevant indices (Supplementary Table 1) for the generation of high genomic merit calves. Cells from the FL065 cell line were subsequently used as donor cells in SCNT. The *in vitro* embryo development rate on day 7 was 23.7% (49/204, Supplementary Table 2), which is comparable to *in vitro* development commonly observed in bovine SCNT with fibroblasts^11^. On day 7, high quality embryos were selected and transferred individually to 16 synchronized recipient cows to produce calves. Pregnancy initiation at 40 days of gestation was 69% (11/16). Thereafter, four pregnancies were lost between 40 and 60 days and the remaining pregnancies continued through gestation to produce 7 calves. Five of these calves were healthy (Fig. 1b). One calf was born about 10 days prematurely and another calf had weakness on the second day of birth; both of these calves died a few days after birth.

DNA samples isolated from ear skin biopsy samples from the live calves were submitted for genomic analysis to validate concordance of genotypes with the genotype of the original cell line, FL065. Comparison of the genomic analysis confirmed high concordance (>99.4%) between FL065 and the live calves produced using these methods (Fig. 1c). In contrast, we detected poor concordance (~60%) between either FL065 or live calves and FL053 and FL070, which were cell lines determined to have poor genetic merit (Fig. 1c). We conclude that GS can be combined with reproductive technologies and SCNT to rapidly and robustly produce calves of a desired genotype.

This study evaluated a method for acceleration of genetic gain in cattle breeding programs by combining GS with advanced reproductive technologies and is, to our knowledge, the first report of production of calves with high genetic merit using an approach that substantially reduces generation interval. Collection of early stage embryos after multiple embryo transfer into synchronized recipient cows and the establishment of cell lines from these embryos allowed rapid determination of enhanced genetic merit for a large number of candidate embryos. Fibroblast cell lines established from early stage embryos supported the production of high genetic merit calves by SCNT with efficiency comparable to IVF embryos. This method reduces the generation interval by approximately 7 months and offers the chance to produce multiple animals at the same or later time from banked, frozen fibroblast cell lines. In contrast, pre-implantation stage embryo biopsy allows the production of only one animal. Moreover, we estimate that selection of high genetic merit at an early embryonic stage reduces 40% of the cost of purchasing and managing recipient females and maximizes value by selectively transferring embryos with the desired genotypes. We expect that the application of this method will substantially improve genetic gain in GS-based animal breeding programs and may help meet projected global demand for milk and meat production.

## Methods

All *in vivo* procedures in this study were carried out in accordance with International Embryo Transfer Society guidelines^12^. Experimental procedures involving animals were performed according to established standard operating procedures for agricultural animal production, which were approved by the Trans Ova Institutional Animal Care and Use Committee. All procedures involving animals were overseen by a licensed veterinarian.

### OPU

Oocytes were retrieved as previously described^13^ using an ultrasound scanner equipped with a 5 MHz linear array transducer. A 50-cm long 17-gauge needle was attached to Tygon tubing leading to the oocyte collection filter. An aspiration pump was used to create a vacuum of 60–70 mmHg, generating a fluid flow of 20–25 ml/min. Donor cows received 4–6 ml of lidocaine (Aspen Veterinary products, Liberty, MD) in the caudal epidural space prior to OPU. Oocytes were collected in Vigro Flushing Solution (Bioniche Animal Health, Pullman, WA). After aspirating the follicles, the filter was washed and the contents poured into a square grid dish and oocytes were located using a stereomicroscope. High quality oocytes were selected based on the cumulus investment and morphology for IVF^14^.

### *In vitro* embryo production

The technique for *in vitro* embryo production has been described in detail previously^15^. Briefly, collected oocytes were washed twice in Vigro Holding Plus Medium (Bioniche Animal Health) and transferred to maturation medium (TCM 199 [Sigma, St. Louis, MO], 10% fetal calf serum [Hyclone, Logan, UT], 0.01 IU/ml bFSH [Sioux Biochem, Sioux Center, IA], 0.01 IU/ml bLH [Sioux Biochem], estradiol 1 μg/ml [Sigma] and antibiotics [penicillin G sodium 50 IU/ml and streptomycin 50 μg/ml]). Oocytes were matured in 400 μl of maturation medium in 6-well plates in 5% CO_2_ in humidified air at 39°C for 20–24 h. At the end of the maturation period, the oocytes were washed twice in pre-incubated fertilization medium in a 6-well plate (500 μl per well). Frozen semen from a Jersey bull was thawed at 37°C for 30 s and layered on Isolate density gradient medium (Irvine Scientific, Irvine, CA) in a 15 ml tube and centrifuged at 700 × g for 15 min to select the live and motile sperm suspension. 20 μl of sperm suspension with a final concentration of 10^6^ sperm/ml was added to each fertilization well. After 18 h of co-incubation, oocytes were vortexed to remove the cumulus and sperm cells, washed once in SOF culture medium and cultured for 7 d in SOF containing 6-well plates under mineral oil in an incubator set up with 5% O_2_ and 5% CO_2_ air at 38.5°C.

### Embryo transfer

Day 7 grade 1 and 2 (Ref. 11) *in vitro* produced embryos derived from oocytes retrieved from donor Jersey cows and *in vitro* fertilized with Jersey sperm were selected and loaded 3–6 embryos per straw and transferred non-surgically into day 7 synchronized recipients.

### Collection of embryos

Embryos were collected on 20–26 d of gestation using a modification of a previously described method^16^. Recipient cows were confined in a cattle chute and given an epidural block of 4–6 ml lidocaine. A sterile 20-gauge Foley catheter was inserted through the cervix into entrance of a horn near the uterine body and the cuff was inflated to keep the catheter in position. Then Vigro Complete Flushing Solution (Bioniche Animal Health) was flushed through the uterus non-surgically while gently squeezing out from the horn of the uterus towards the cervix to expel the fetuses and membranes with the flushing medium. This flushing procedure was then repeated on the contralateral uterine horn. The flushed medium was collected via gravity flow in an EZ way filter (SPI, Canton, TX). The flushed contents and filter were taken to the laboratory and carefully washed onto a square grid search dish under a laminar flow hood. The embryos were collected using a stereomicroscope.

### Cell line establishment

Bovine fetal fibroblast cell lines were established using a modification of a method described earlier^17^. Briefly, bovine embryos flushed from the recipient cows were transported to the laboratory in Dulbecco's phosphate buffered saline solution (DPBS) with 16 μl/ml of antibiotic-antimycotic (Life Technologies, Grand Island, NY), and 8 μl/ml fungizone (Life Technologies). Embryos were rinsed in DPBS, the fetal membranes were removed and the whole embryo was disaggregated using a scalpel. Fibroblasts were separated by a standard trypsinization procedure using Tryp-LE (Life Technologies). Cells were seeded onto 25 mm tissue culture flasks (BD Falcon) in DMEM (Life Technologies) supplemented with 10% fetal calf serum (Hyclone, Logan, UT), 0.15 g/ml glutamine (Sigma), and 0.003% β-mercaptoethanol (Life Technologies). On day 4 of seeding, the cells were passaged and reseeded onto 250 mm tissue culture flasks. Cells were harvested and frozen in DMEM with 10% FCS and 10% DMSO (Sigma) once they were 80–90% confluent.

### DNA isolation and genotyping

DNA was extracted from fibroblast cell lines using a standard procedure. Briefly, 90% confluent fibroblasts in 12 mm flasks were lysed using 500 μl cell lysis solution (Qiagen, Valencia, CA) and transferred to a 1.5 ml microcentrifuge tube and incubated at room temperature for 8–12 h. Next, 150 μl of protein precipitation solution was added, the mixture was shaken vigorously and centrifuged at 13,000 rpm at 2–8°C for 3 min in a refrigerated microcentrifuge. The supernatant was transferred to a new tube containing 300 μl of 99.5% isopropanol (Sigma), mixed by gentle inversion of the microcentrifuge tube and centrifuged for 5 min at 13,000 rpm at 2–8°C. The supernatant was discarded and the DNA pellet was washed in 300 μl of 70% ethanol and centrifuged as above. The supernatant was discarded and the pellet was dried at room temperature for 30 min. Then the pellet was dissolved in TE buffer (Sigma). DNA was checked for size and quality on a 1% TAE-agarose gel. Samples were submitted to a commercial laboratory (Neogen GeneSeek, Lincoln, NE) for genotyping using the custom GeneSeek Genomic Profiler (GGP) low-density BeadChip, which utilizes Illumina Infinium chemistry with approximately 10,000 SNPs assayed per sample. Cell line genomic results were submitted to the Council on Dairy Cattle Breeding (CDCB) for genomic enhanced dairy genetic evaluation. Genetic merit values and industry indexes were then used for cell line ranking.

### Nuclear transfer

The nuclear transfer procedure was performed essentially as described previously^18^. Briefly, *in vitro*-matured slaughter house derived oocytes were enucleated about 18–20 h post-maturation. After transferring donor cells into the perivitelline space, embryos were fused, using a single electrical pulse of 2.4 kV/cm for 20 μsec (Electrocell Manipulator 2001, Harvard Apparatus, Holliston, MA). At 26 h, post-maturation reconstructed oocytes were activated with calcium ionophore (5 μM; Cal Biochem, San Diego, CA) for 4 min and 10 μg cycloheximide and 2.5 μg cytochalasin D (Sigma) as described earlier. After activation, cloned embryos were placed in culture in 4-well tissue culture plates, containing 0.5 ml of synthetic oviductal fluid medium covered with mineral oil (Sigma) and incubated at 38.5°C in a 5% O_2_ and 5% CO_2_ in air atmosphere. On day 7, grade (Ref. 11) 1 and 2 embryos were selected and transferred using standard embryo transfer procedures into single embryo per synchronized recipient.

### Concordance analysis

We performed pairwise comparisons between SNP data from different samples in order to determine concordance between sample genotypes. Due to differences in array construction, the number of genotyped loci was not the same for all samples. Concordance analysis was therefore performed only for the loci that were genotyped in both samples of a given pair. Additionally, SNP calls with Illumina GenCall scores, which correlate with SNP call accuracy^19^, less than 0.5 were discarded in order to ensure that comparisons were performed only using loci with high-confidence genotypes. On average, ~16,000 SNPs met these criteria in a given pairwise comparison. Concordance percentages were computed by dividing the number of loci with identical genotypes by the total number of loci meeting the above criteria in a given pairwise comparison.

## Author Contributions

P.K., H.W., M.F.A. and D.C.F. conceived and designed the experiments with contributions from J.M., D.B. and S.K. H.W. produced the IVF embryos, J.A.M. transferred the embryos and collected the fetuses, P.K. selected the fetuses, established cell lines, and extracted DNA. M.F.A. and J.M. submitted the DNA samples for genomic analysis. T.X. produced SCNT embryos. P.K. and S.K. wrote the manuscript and analyzed data with contributions from all authors.

## Supplementary Material

Supplementary Information Supplementary Information

## Figures and Tables

**Figure 1:**
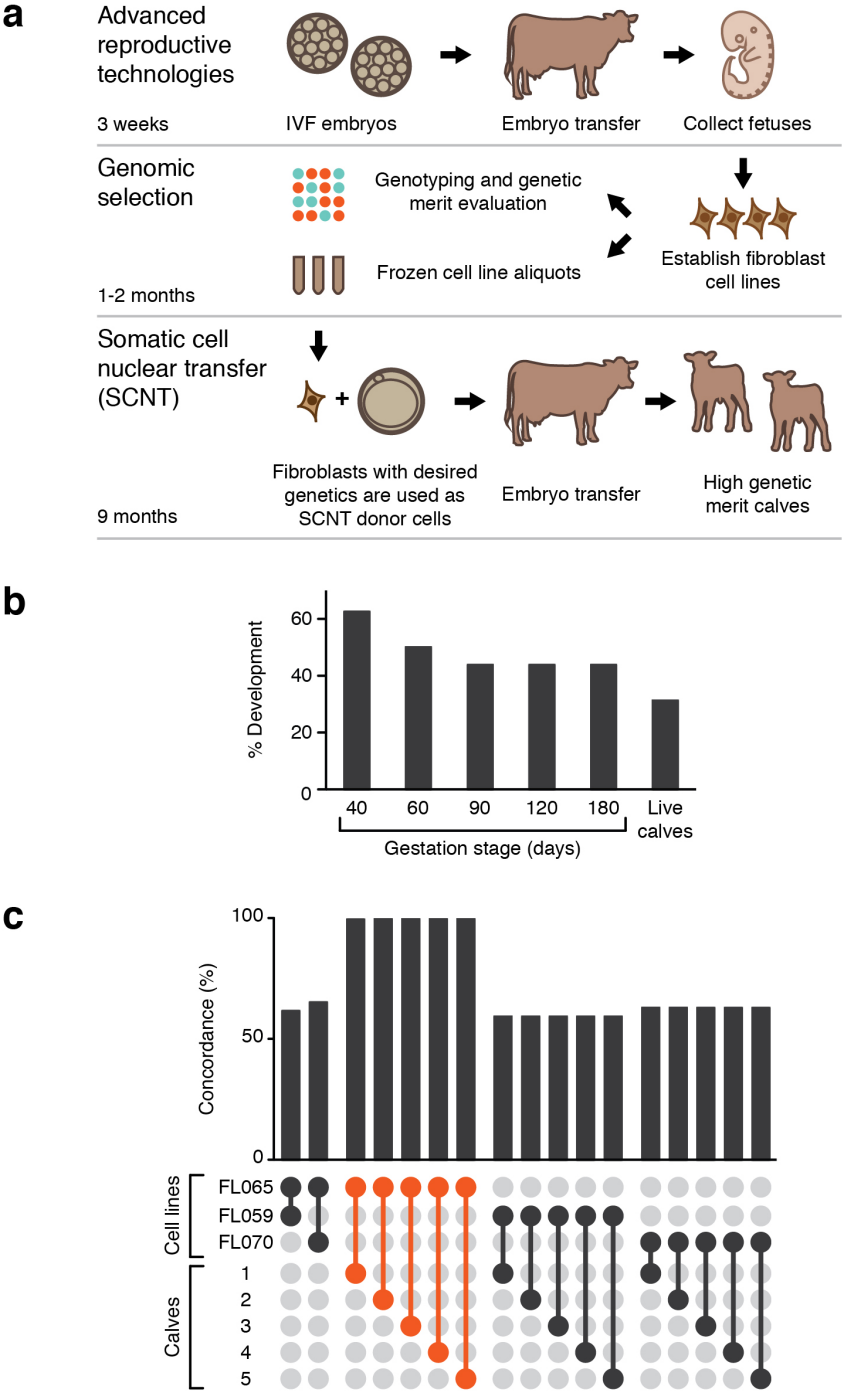
Production of high genetic merit calves. (a) Schematic representation of reproductive processes employed in this study. (b) *In vivo* development of nuclear transfer embryos using donor cells from FL065 cell line. Embryos were transferred into 16 recipient cows. Embryo development rate was calculated from the number of embryos transferred to recipients. (c) Concordance of genotyping results from the cell line FL065, DNA samples collected from the five live calves produced from FL065, and two other randomly selected cell lines, FL059 and FL070, which were derived from IVF-produced fetuses with poor genetic merit.

**Table 1:** Embryo transfer, fetal recovery and cell lines with multiple embryo transfers

Gestation stage at fetal collection (days)	Recipients transferred	Embryos transferred	Fetuses collected (%)	Useable cell lines established (%)
21–23	11	70	43 (61.4)	43 (100)
24–26	20	119	62* (50.4)	60 (96.8)

(Fisher exact test, p > 0.36)

*Two of the cell lines had fungal contamination and were discarded.
