# Supplementary material for: Acceleration of genetic gain in cattle by reduction of generation interval
Source: Sci Rep. 2015 Mar 2;5:8674. doi: 10.1038/srep08674 (PMC4345332; doi:10.1038/srep08674)
Supplement: Supplementary Information [file srep08674-s1.pdf]

Acceleration of genetic gain in cattle by reduction of generation interval

Poothappillai Kasinathan[1*], Hong Wei[1], Tim Xiang[1], Jose Molina[1], John Metzger[1], Diane Broek[1], Sivakanthan Kasinathan[2], David C. Faber[1] and Mark F. Allan[1]

[1]Trans Ova Genetics, LC, Sioux Center, IA 51250.
[2]Medical Scientist Training Program, University of Washington School of Medicine, Seattle, WA 98195.
*Corresponding author: P. Kasinathan (kasi@transova.com)

Supplementary Materials

**Supplementary Table 1**	Genomic enhanced genetic merit analysis of fibroblast cell lines. Values for industry indexes commonly used to evaluate genetic merit of cattle.
**Supplementary Table 2**	*In vitro* development of embryos derived from fibroblast cell lines.

Supplementary Table 1: Genomic analysis of DNA from cell lines

Cell line ID	JPI Gen	CM Gen	NM Gen	PTAT Ge	MILK Ge	FAT Gen	PRO Gen	PL Gen	SCS Gen	DPR Gen	UDC Gen	Haplotypes
**FL065**	251	750	680	2.1	880	75	41	6.5	2.86	1.2	6.13	JH1T JH1_PC.N JHPT JHP_PC.N
**FL062**	224	688	623	1.8	669	65	34	6.2	2.86	1.6	5.02	JH1T JH1_PC.N JHPT JHP_PC.N
**FL064**	221	708	618	1	501	63	33	6	2.73	1.5	4.92	JH1T JH1_PC.N JHPT JHP_PC.N
**FL017**	215	692	607	0.9	470	60	31	5.8	2.72	1.9	4.35	JH1T JH1_PC.N JHPT JHP_PC.N
**FL036**	210	594	571	1.4	909	32	29	6.6	2.76	1.1	5.76	JH1T JH1_PC.N JHPT JHP_PC.N
**FL066**	205	673	598	1.7	465	63	28	6.3	2.72	0.2	5.56	JH1T JH1_PC.N JHPT JHP_PC.N
**FL040**	192	531	484	1.1	782	38	32	4.3	2.8	1	5	JH1T JH1_PC.N JHPT JHP_PC.N
**FL030**	192	507	509	1.5	1191	33	32	5.6	2.8	1.1	5.22	JH1T JH1_PC.N JHPT JHP_PC.N
**FL067**	191	646	565	1.2	237	58	24	6.1	2.78	1.2	4.81	JH1T JH1_PC.N JHPT JHP_PC.N
**FL038**	185	562	499	1.3	507	40	28	4.8	2.81	1.2	4.43	JH1T JH1_PC.N JHPT JHP_PC.N
**FL022**	184	508	490	1	1313	50	40	3.7	2.77	0.2	1.78	JH1T JH1_PC.N JHPT JHP_PC.N
**FL004**	183	546	484	1.5	520	46	28	4.2	2.8	0.4	4.9	JH1T JH1_PC.N JHPT JHP_PC.N
**FL039**	180	456	432	0.8	1240	45	41	3.4	2.89	-0.1	2.88	JH1T JH1_PC.N JHPT JHP_PC.N
**FL009**	176	460	432	1.1	1067	30	36	4.1	2.87	0	3.89	JH1T JH1_PC.N JHPT JHP_PC.N
**FL010**	176	524	456	1.8	474	47	28	3.7	2.79	0.4	5.2	JH1T JH1_PC.N JHPT JHP_PC.N
**FL056**	174	593	526	0.4	490	59	27	4.6	2.73	1	2.51	JH1T JH1_PC.N JHPT JHP_PC.N
**FL058**	174	593	526	0.4	490	59	27	4.6	2.73	1	2.51	JH1T JH1_PC.N JHPT JHP_PC.N
**FL033**	174	499	463	1.4	809	35	30	4.1	2.82	1	3.62	JH1T JH1_PC.N JHPT JHP_PC.N
**FL063**	173	566	512	1.4	317	50	20	5.5	2.79	1.5	4.69	JH1T JH1_PC.N JHPT JHP_PC.N
**FL001**	171	453	453	1	1109	31	30	4.2	2.82	0.6	3.17	JH1T JH1_PC.N JHPT JHP_PC.N
**FL042**	170	564	497	0.1	429	33	26	5.2	2.8	1.9	2.82	JH1T JH1_PC.N JHPT JHP_PC.N
**FL053**	170	458	465	1.5	1011	24	24	5.4	2.75	1.2	5.25	JH1T JH1_PC.N JHPT JHP_PC.N
**FL011**	169	484	423	0.9	517	29	28	4.2	2.84	1.1	5.03	JH1T JH1_PC.N JHPT JHP_PC.N
**FL054**	168	585	496	0.2	45	47	19	4.8	2.67	1.9	3.55	JH1T JH1_PC.N JHPT JHP_PC.N
**FL055**	168	585	496	0.2	45	47	19	4.8	2.67	1.9	3.55	JH1T JH1_PC.N JHPT JHP_PC.N
**FL043**	165	525	459	0.3	474	31	26	4.7	2.71	1.5	2.79	JH1T JH1_PC.N JHPT JHP_PC.N
**FL046**	165	525	459	0.3	474	31	26	4.7	2.71	1.5	2.79	JH1T JH1_PC.N JHPT JHP_PC.N
**FL029**	164	432	447	1.7	994	33	23	5.1	2.79	0.9	5.21	JH1T JH1_PC.N JHPT JHP_PC.N
**FL018**	161	434	435	1.8	850	36	22	4.9	2.8	1	5.5	JH1T JH1_PC.N JHPT JHP_PC.N
**FL006**	158	453	403	0.8	635	34	29	3.1	2.85	0.7	3.06	JH1T JH1_PC.N JHPT JHP_PC.N
**FL016**	155	510	444	0.9	261	42	21	4.6	2.81	1.2	3.53	JH1T JH1_PC.N JHPT JHP_PC.N
**FL008**	154	462	422	0.9	404	33	21	4.3	2.92	1	4.07	JH1T JH1_PC.N JHPT JHP_PC.N
**FL068**	153	487	450	0.8	559	49	23	4.6	2.85	0.4	3.01	JH1T JH1_PC.N JHPT JHP_PC.N
**FL037**	153	391	390	1.1	917	22	26	4.1	2.92	1	3.61	JH1T JH1_PC.N JHPT JHP_PC.N
**FL069**	151	477	431	0.9	582	38	25	4.1	2.77	0.9	2.72	JH1T JH1_PC.N JHPT JHP_PC.N
**FL057**	151	523	459	0.2	255	56	21	4	2.81	1.5	1.91	JH1T JH1_PC.N JHPT JHP_PC.N
**FL052**	150	455	450	1.7	543	25	13	5.8	2.66	1.2	5.28	JH1T JH1_PC.N JHPT JHP_PC.N
**FL045**	149	518	488	0.2	389	29	16	6.3	2.8	1.7	2.73	JH1T JH1_PC.N JHPT JHP_PC.N
**FL005**	149	435	392	1.2	478	36	23	3.3	2.89	0.6	3.83	JH1T JH1_PC.N JHPT JHP_PC.N
**FL070**	149	457	418	1.2	448	46	21	3.8	2.88	0.8	4.23	JH1T JH1_PC.N JHPT JHP_PC.N
**FL032**	140	426	380	1.1	417	30	21	3.5	2.83	1	3.37	JH1T JH1_PC.N JHPT JHP_PC.N
**FL007**	139	364	348	1.1	794	21	26	3	2.9	0.5	3.03	JH1T JH1_PC.N JHPT JHP_PC.N
**FL060**	138	489	438	0.3	318	40	19	4.5	2.75	1.1	1.23	JH1T JH1_PC.N JHPT JHP_PC.N
**FL061**	138	489	438	0.3	318	40	19	4.5	2.75	1.1	1.23	JH1T JH1_PC.N JHPT JHP_PC.N
**FL014**	138	478	434	0.4	252	33	15	5.3	2.79	1.5	2.45	JH1T JH1_PC.N JHPT JHP_PC.N
**FL023**	134	325	354	1.4	1097	17	23	4.2	2.83	0.5	3.9	JH1T JH1_PC.N JHPT JHP_PC.N
**FL025**	134	325	354	1.4	1097	17	23	4.2	2.83	0.5	3.9	JH1T JH1_PC.N JHPT JHP_PC.N
**FL073**	134	461	393	1.1	207	29	19	3.7	2.72	1.3	2.27	JH1T JH1_PC.N JHPT JHP_PC.N
**FL035**	132	452	397	1.2	18	15	10	4.9	2.74	1	5.14	JH1T JH1_PC.N JHPT JHP_PC.N
**FL034**	132	337	306	1.1	464	22	20	2.8	2.87	0	5.04	JH1T JH1_PC.N JHPT JHP_PC.N
**FL050**	131	331	339	1.2	904	26	22	3.5	2.82	0.8	3.87	JH1T JH1_PC.N JHPT JHP_PC.N
**FL048**	131	383	402	1.2	605	10	10	6.1	2.77	1.4	4.69	JH1T JH1_PC.N JHPT JHP_PC.N
**FL020**	129	404	392	1.3	433	23	13	5.2	2.77	1	4.87	JH1T JH1_PC.N JHPT JHP_PC.N
**FL013**	128	443	395	0.7	40	22	11	5.1	2.87	1.9	3.87	JH1T JH1_PC.N JHPT JHP_PC.N
**FL051**	122	384	396	1	610	5	11	6.1	2.69	1.1	3.54	JH1T JH1_PC.N JHPT JHP_PC.N
**FL041**	121	422	362	0.2	141	30	17	4	2.91	1.1	1.98	JH1T JH1_PC.N JHPT JHP_PC.N
**FL026**	121	314	340	1	766	18	14	4.7	2.84	1.1	3.94	JH1T JH1_PC.N JHPT JHP_PC.N
**FL024**	119	322	336	1	824	15	18	4.4	2.8	0.7	3.15	JH1T JH1_PC.N JHPT JHP_PC.N
**FL049**	119	293	308	1.1	883	12	21	4.1	2.9	0.6	3.38	JH1T JH1_PC.N JHPT JHP_PC.N
**FL031**	119	324	288	1.1	452	20	20	2.2	2.86	0.3	3.61	JH1T JH1_PC.N JHPT JHP_PC.N
**FL019**	116	329	321	1.3	558	17	17	4.4	2.95	0.7	4.15	JH1T JH1_PC.N JHPT JHP_PC.N
**FL044**	116	442	389	-0.1	220	30	16	4.3	2.75	1	0.68	JH1T JH1_PC.N JHPT JHP_PC.N
**FL047**	116	442	389	-0.1	220	30	16	4.3	2.75	1	0.68	JH1T JH1_PC.N JHPT JHP_PC.N
**FL072**	112	372	312	0.5	177	26	16	2.6	2.71	0.7	2.37	JH1T JH1_PC.N JHPT JHP_PC.N
**FL059**	110	398	344	-0.2	133	20	15	3.9	2.82	1.3	0.88	JH1T JH1_PC.N JHPT JHP_PC.N
**FL071**	75	272	235	0.4	186	7	12	2.8	2.78	0.8	0.58	JH1T JH1_PC.N JHPT JHP_PC.N

JPI- Jersey performance Index; CM-Cheese Merit; NM-Net Merit; PTAT- ;PRO-Protein; PL- ;SCS-Somatic Cell Score; DPR-Daughter Pregnancy Rate; UDC- Udder Cleft and Gen- General

Supplementary Table 2: *In vitro* development of embryos from established cell lines

Cell line ID	Total nuclear transfers	Number of fused couplets (%)	Number of blastocyst on day 7 (%)*
FL010	327	222 (67.9)	40 (18.0)
FL065	321	207 (64.5)	49 (23.7)
Total	648	429 (65.2)	89 (20.8)

*Blastocysts development rate was calculated from number of oocytes fused with donor somatic cell.
